# IMB-T130 targets 3-dehydroquinate synthase and inhibits Mycobacterium tuberculosis

**DOI:** 10.1038/s41598-018-35701-z

**Published:** 2018-11-28

**Authors:** Ningyu Zhu, Xia Wang, Dongsheng Li, Yuan Lin, Xuefu You, Jiandong Jiang, Yanni Xu, Wei Jiang, Shuyi Si

**Affiliations:** 1 Beijing Key Laboratory of Antimicrobial Agents, Institute of Medicinal Biotechnology, Peking Union Medical College and Chinese Academy of Medical Sciences, Beijing, 100050 China; 2 State Key Laboratory of Bioactive Substances and Function of Natural Medicine, Institute of Materia Medica, Chinese Academy of Medical Sciences and Peking Union Medical College, Beijing, 100050 China

## Abstract

The anti-tuberculosis (TB) agent IMB-T130 was speculated to be a multi-target compound. In this research, we found that IMB-T130 inhibits the catalytic activity of *Mycobacterium tuberculosis* 3-dehydroquinate synthase (*Mt*DHQS), the enzyme in the second step of the shikimate pathway. IMB-T130 was identified as a selective inhibitor of *Mt*DHQS with an IC_50_ value of 0.87 μg/mL. The interaction between the compound and protein was analysed by surface plasmon resonance and circular dichroism. Based on the *in silico* molecular docking results, the essential amino acids in the binding pocket were then confirmed by site-directed mutagenesis. Overexpression of DHQS reduced the antibacterial activity of IMB-T130 in cells, verifying that DHQS is the target of IMB-T130. IMB-T130 inhibited standard and drug-resistant *M. tuberculosis* strains by targeting DHQS. Our findings improve our understanding of *Mt*DHQS and make it to be a potential target for new anti-TB drug discovery.

## Introduction

Tuberculosis (TB), a serious infectious disease caused by *Mycobacterium tuberculosis*, is a major threat to global human health. The latest data from the World Health Organization’s Global Tuberculosis Report shows that TB killed 1.5 million people in 2014, and about 9.6 million people are estimated to be infected with *M. tuberculosis* worldwide^1^. TB is commonly treated with a combination of several antibiotics, but the long and complicated treatment protocols increase the probability of drug-resistant bacterial strains emerging. Although the anti-TB agents bedaquiline and delamanid have now been introduced to the market^2^, there remains an urgent need to discover new anti-TB drugs that can shorten the treatment period and overcome the growing problem of drug resistance.

Chorismic acid, which is synthesized through the shikimic acid pathway, is the precursor of aromatic amino acids and other metabolites, including folates, ubiquinones, mycobactins, menaquinones, and napthoquinones^3^. Genomic studies have shown that this biosynthetic route is essential in bacteria, fungi, algae, higher plants, and some parasites, such as the human malaria-causing parasite *Plasmodium falciparum*^4–6^. The essential role of the shikimic acid pathway in these organisms has been confirmed by site-directed mutagenesis, where the growth of mutant strains was inhibited in the absence of exogenous aromatic supplements^7^. The absence of this pathway in mammals makes it an attractive target for developing new antibiotics with low toxicity toward humans^4^.

*M. tuberculosis* 3-dehydroquinate synthase (DHQS), which is encoded by the *aroB* gene (Rv2538c), catalyses the second step of the shikimate pathway. It catalyses the conversion of 3-deoxy-D-Darabino-heptulosonate 7-phosphate (DAHP) to 3-dehydroquinate (DHQ)^8,9^. NAD^+^ acts as a cofactor in this reaction, which comprises oxidation, β-elimination, intramolecular aldol condensation, and reduction^10,11^. The *aroB* gene was revealed to be essential by transposon site hybridization (TraSH) in 2003^12,13^. Based on this finding, Sassetti *et al*. and Griffin *et al*. also found that the *aroB* gene is essential using similar deep sequencing methods^13,14^.

**Figure 1:**
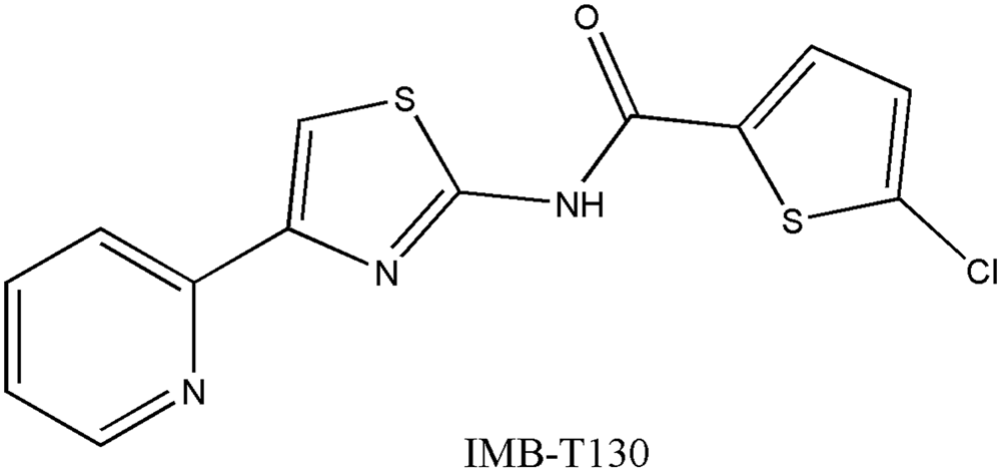
Structure of IMB-T130 (5-chloro-N-[4-(pyridin-2-yl)-1, 3-thiazol-2-yl] thiophene-2-carboxamide).

We reported previously that IMB-T130 is a potential selective anti-TB agent, based on its excellent inhibitory activity against drug-resistant TB^15^. In the present study, we found that IMB-T130 strongly inhibited DHQS *in vitro* (Fig. 1), and that DHQS may play an important role in the antibacterial effect of IMB-T130.

## Results

### Expression and purification of DHQS

The DHQS recombinant protein was purified using Ni^2+^ His Trap chelating columns and the purification was confirmed after SDS-PAGE and Coomassie blue staining. Only a single band appeared at 38 kDa (Supplementary Fig. 1). DHQS catalyses the conversion of DAHP to 3-dehydroquinate (DHQ) with NAD^+^, and releases a molecule of free phosphoric acid. The catalytic activity of DHQS was tested by measuring phosphoric acid using malachite green solution (Biomol Green®).

### Screening for compounds that inhibit DHQS

**Figure 2:**
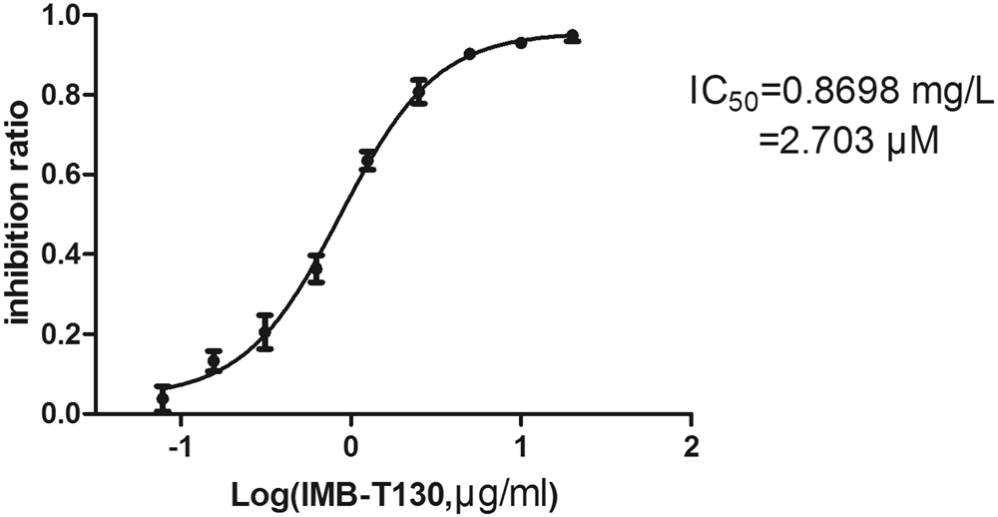
Inhibition of DHQS by IMB-T130. DHQS (1 μg/mL) was incubated with 0.15–20 μg/mL of IMB-T130 at room temperature for 30 min and the catalytic activity was determined (see Methods section). The IC_50_ value is plotted as the inhibition ratio of the OD_630_ over the compound concentration (log plot) that fits a variable-slope dose-response equation. The experiment was repeated three times.

We have previously screened a library containing 20,000 compounds using *Mycobacterium smegmatis*, which is a nonpathogenic species that is biologically close to *M. tuberculosis*^15^. Based on this result, we searched for DHQS inhibitors using an enzyme assay. IMB-T130 was the only compound with an OD_630_ of less than 50% of the control (1% DMSO) at 10 μg/mL, we inferred that it may have inhibitory activity towards DHQS. Therefore, the inhibition ratios of different concentrations of IMB-T130 (20, 10, 5, 2.5, 1.25, 0.625, 0.31, and 0.16 μg/mL) were determined. The activity of *Mt*DHQS was inhibited by IMB-T130 in a dose-dependent manner, with an IC_50_ value of 0.8698 μg/mL (2.703 μM) (Fig. 2).

### Confirming the interaction between IMB-T130 and DHQS

**Figure 3:**
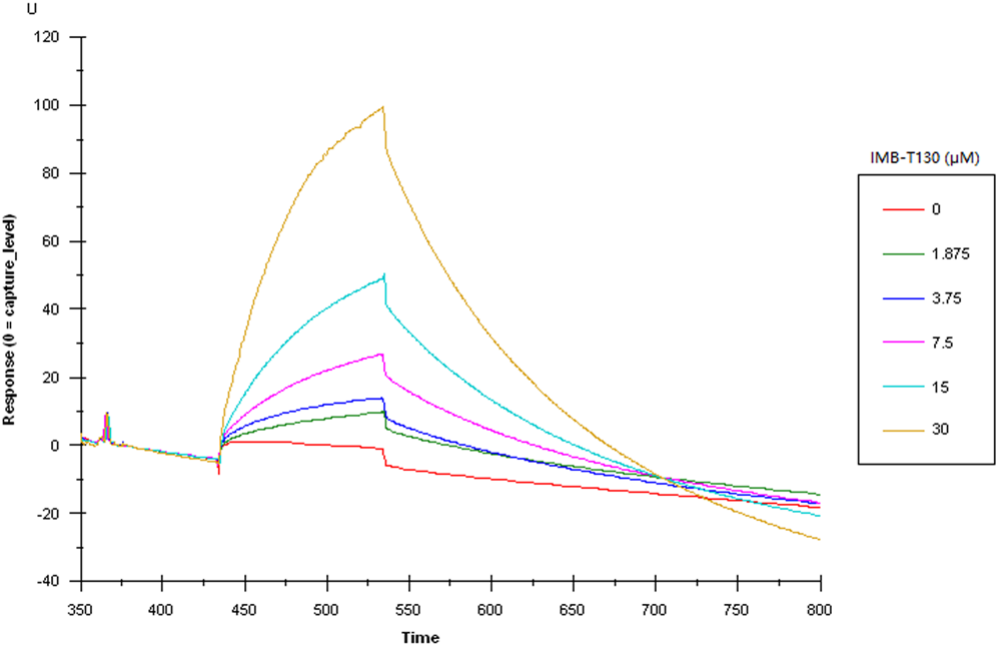
SPR results for the interaction between DHQS and IMB-T130. A sensor chip coated with purified protein was exposed to 1.875–30 μM of IMB-T130. The change in response units is shown.

Surface plasmon resonance (SPR) could detect interactions between compounds and proteins. DHQS protein is unstable under acidic conditions, so the combination of IMB-T130 and DHQS was detected using nitrilotriacetic acid (NTA) sensor chip. The chips were coated with Ni^+^ to capture the His-tagged protein and then exposed to a concentration gradient (1.875–30 μM) of IMB-T130. The response units were determined by SPR^16,17^. The result showed a significant dose-dependent response for the concentrations tested (Fig. 3).

The circular dichroism (CD) spectrum of DHQS showed significant secondary structure changes after binding IMB-T130^18^. The differences between the native protein and the complex are reflected in the composition of the α-helix (71.6% vs 63.3%), β-turn (11.2% vs 12.4%), and random coil (13.4% vs 16.8%) (Supplementary Fig. 2).

### Molecular docking between IMB-T130 and DHQS

**Figure 4:**
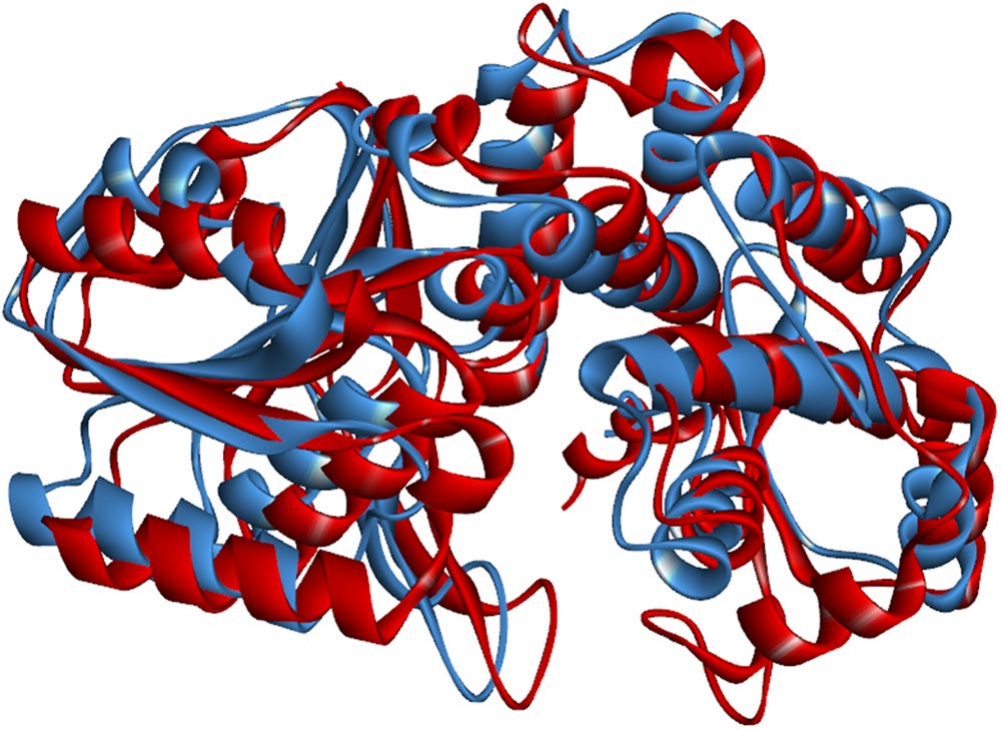
Superposition of *Mt*DHQS (red) and *Sa*DHQS (blue).

The crystal structure of *Mt*DHQS solved at 2.07 Å resolution was retrieved from the Protein Data Bank (PDB ID code 3QBE), and we established an initial model based on the structural data. The key amino acids were not specific because of the lack of substrate molecules. Therefore, using the crystal structure of *Sa*DHQS complexed with its substrate, we optimized the *Mt*DHQS model and speculated on the key amino acids (Fig. 4).

**Figure 5:**
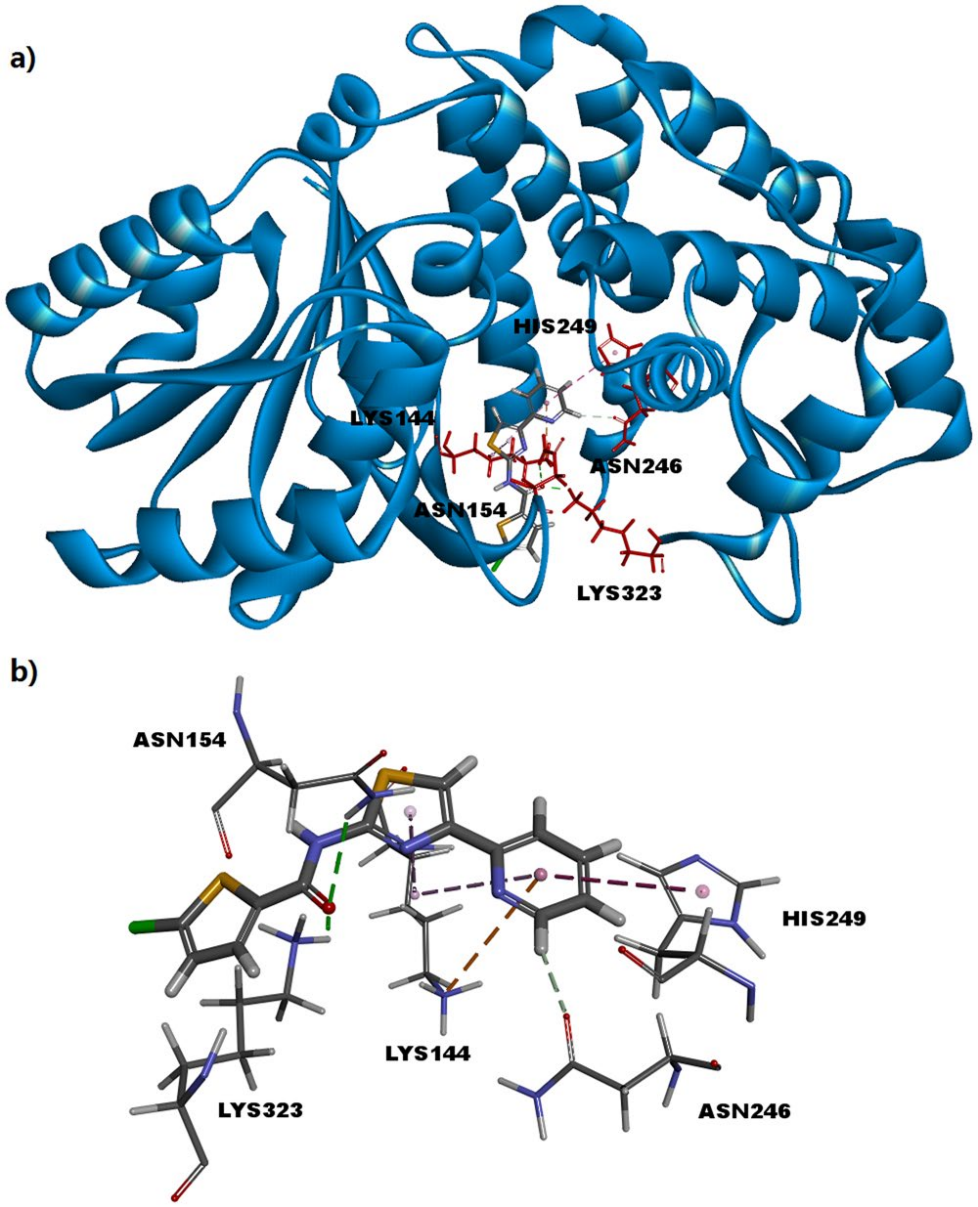
Molecular docking for DHQS and IMB-T130. (**a**) Overview of the active pocket of DHQS bound to IMB-T130. IMB-T130 is represented by a stick model (grey, carbon atoms; blue, nitrogen atoms; red, oxygen atoms; yellow, sulfur atoms; green, chlorine atoms). Red sticks represent the amino acid residues interacting with IMB-T130. (**b**) A carbonyl oxygen atom forms a conventional hydrogen bond with Asn154 and Lys323 (green line). A carbon-hydrogen bond formed between the carbonyl group of Asn246 and pyridine (grey line). Thiazole and pyridine formed different hydrophobic interactions with Lys144, Asn246, and His249 (purple and orange lines).

To analyse the interaction between IMB-T130 and DHQS, molecular docking was performed based on the optimized protein model. The most probable docking model with the highest score for both -CDOCKER energy and -CDOCKER interaction energy showed the compound interacting with five important amino acids in the DHQS active centre (Fig. 5a). A carbonyl oxygen atom formed conventional hydrogen bonds with Asn154 and Lys323. A carbon-hydrogen bond between the carbonyl group of Asn246 and pyridine was observed. In addition, thiazole and pyridine formed different hydrophobic interactions with Lys144, Asn246, and His249 (Fig. 5b).

### Construction of DHQS mutants and measurement of catalytic activity

**Figure 6:**
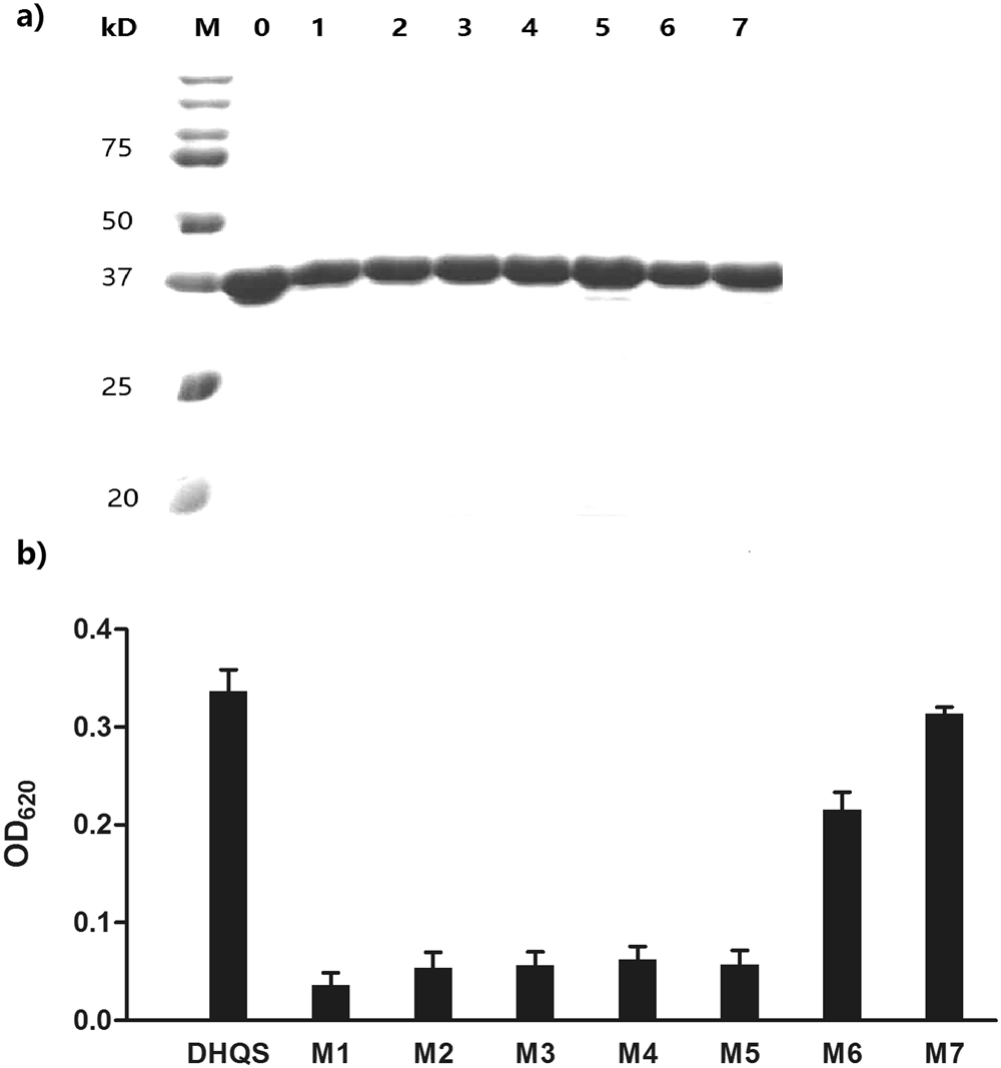
(**a**) Expression and purification of recombinant *M. tuberculosis* DHQS and its mutants. Lane M, protein marker. Lane 0: purified His-tagged *Mt*DHQS. Lanes 1–7: purified mutant proteins DHQS-M1–7. (**b**) Catalytic activity of wild-type DHQS and DHQS-M1–7 at 1 μg/mL. The OD_620_ values represent the progress of the catalytic reaction.

According to the *A. nidulans* DHQS crystal structure data, Lys144, Lys154, Asn246, and Lys323 take part in the β-elimination of the phosphate group. Asn246 is also involved in ring opening and intramolecular aldol condensation, while His249 may interact with Zn^2+ ^^19^. Based on this information, we conducted site-directed mutagenesis to replace Lys144, Asn154, Asn246, His249, and Lys323 with Ala, separately. For the controls, Leu30 and Val40 were also changed to Ala. The mutant proteins were named DHQS-M1 (Lys144), DHQS-M2 (Asn154), DHQS-M3 (Asn246), DHQS-M4 (His249), DHQS-M5 (Lys323), DHQS-M6 (Leu30, Ala control) and DHQS-M7 (Val40, Ala control) (Fig. 6a). After determining the catalytic activities of the mutant enzymes at 1 μg/mL, the results showed that the activities of DHQS-M1–5 were significantly reduced. The activity of mutant protein DHQS-M6 was slightly reduced, whereas the activity of DHQS-M7 remained unchanged. This result indicates that the five amino acids in the active center of the enzyme are indispensable for its structure and function. Leu30 of the α1 helix is not involved in the catalytic reaction process directly, but it may contribute to the maintenance of the protein structure. The mutation of Val40 in the β1 sheet did not affect the function of *Mt*DHQS significantly (Fig. 6b).

### Pharmacokinetic studies of IMB-T130

**Figure 7:**
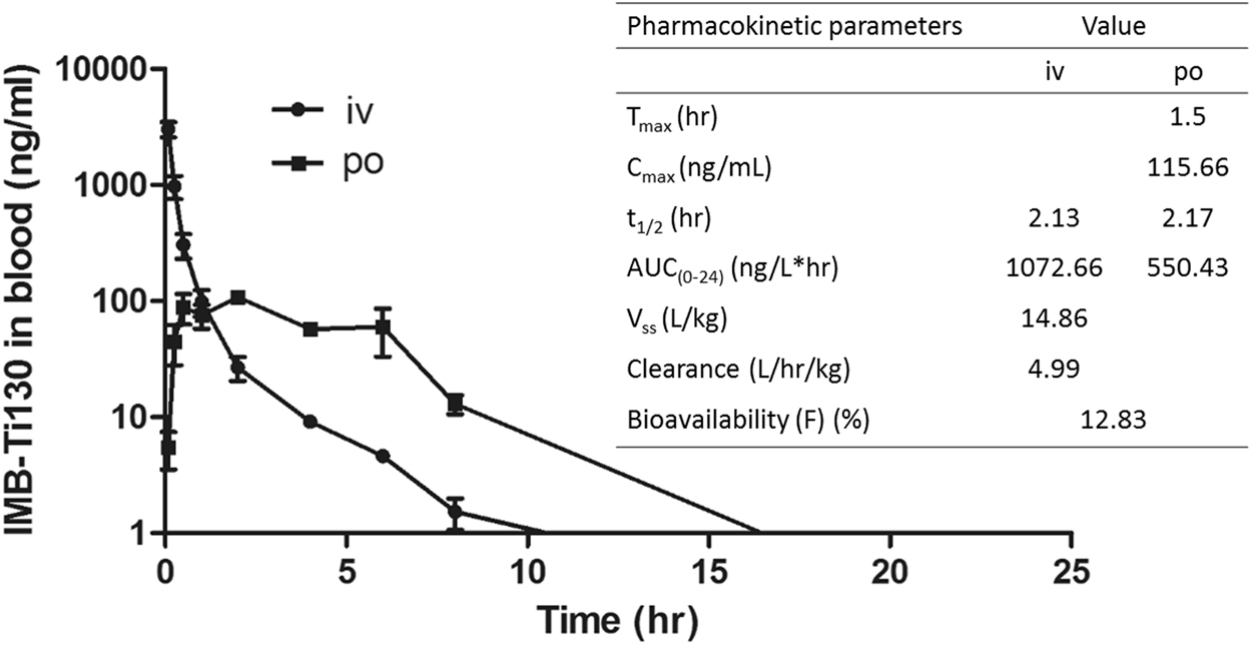
Mean blood concentration profiles of IMB-T130 following oral (p.o) and iv doses of 20 and 5 mg/kg in in male Sprague Dawley rats. Pharmacokinetic parameters were determined after the administration of the oral and iv dose to rats. *T*_*max*_, time to reach *C*_max_; *C*_*max*_, maximum plasma concentration; *t*_*(1/2)*_, half-life; AUC, area under the concentration curve; *V*_*ss*_, volume of distribution at steady state.

Pharmacokinetic parameters were determined after administration of single oral and intravenous (iv) doses of IMB-T130 at 20 and 5 mg/kg, respectively, in male Sprague Dawley rats. The pharmacokinetic parameters showed that IMB-T130 had moderate total plasma clearance (4.99 L/hr/kg), a large volume of distribution (14.86 L/kg), and oral bioavailability 12.83% (Fig. 7).

After oral administration, maximum plasma concentration (*C*_max_) of IMB-T130 reached 115.66 ng/mL at 1.5 h, mouse exposure was higher than the minimum inhibitory concentration MIC (80 ng/mL) for about 4 hours. We speculated that the compound might have anti-TB activity *in vivo*. IMB-T130 was well tolerated in ICR mice at up to 500 mg/kg administered orally. No adverse effects were observed over 72 hours, which indicated that oral administration of IMB-T130 may be safe.

### Higher MIC for the DHQS-overexpression strain

**Figure 8:**
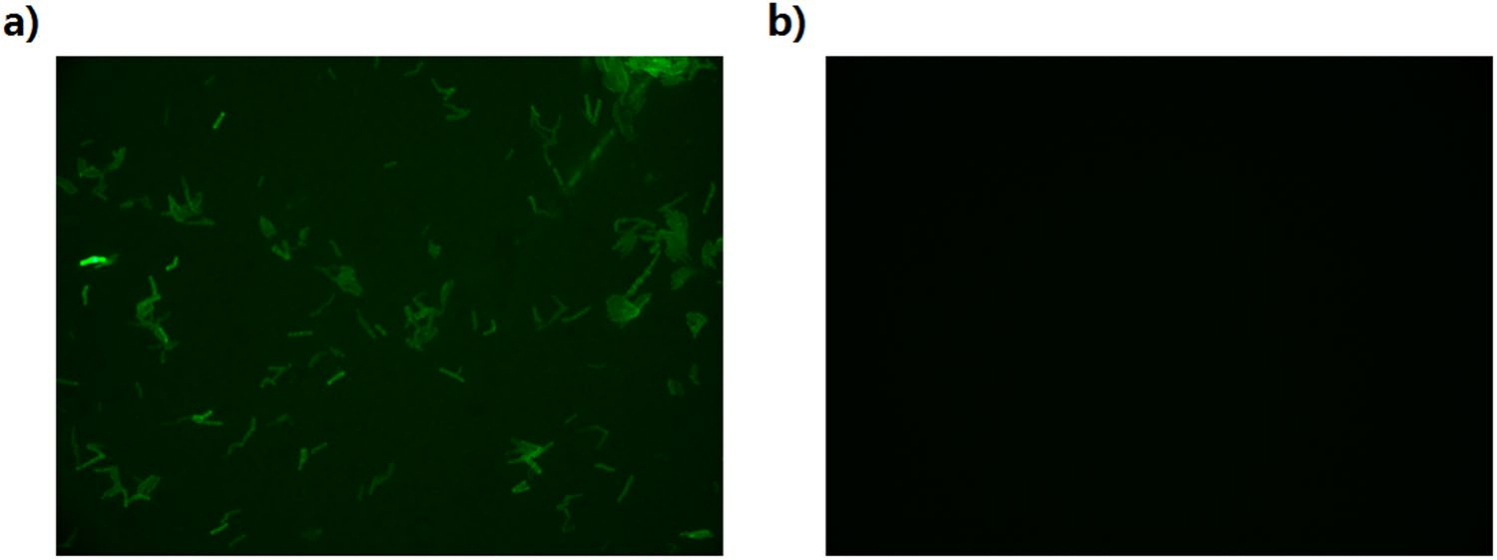
Fluorescence signal in *M. smegmatis* strains with overexpression plasmids for DHQS-eGFP. (**a**) *M. smegmatis* cells carrying plasmid pMV261:DHQS-eGFP, and (**b**) *M. smegmatis* cells carrying plasmid pMV261.

The antibacterial activity of IMB-T130 should be lower for the strains overexpressing DHQS protein. Therefore, we constructed a plasmid containing DHQS-eGFP, which was transformed into *M. smegmatis*^15^. The expression of recombinant DHQS was confirmed by the appearance of a GFP signal (Fig. 8a), while the strain transformed with an empty plasmid showed no signal (Fig. 8b). The MIC for IMB-T130 against the *M. smegmatis* strain transformed with the PMV261 control plasmid was 0.312 μg/mL. In contrast, the MIC of IMB-T130 was 2.5 μg/mL against the DHQS-overexpression strain, which is eight-fold higher than the MIC value for IMB-T130 compared with the control strain (Table 1). *M. tuberculosis* ribosomal protein L12 (*Mt*L12) is not related to this study. Thus, the *M. smegmatis* strain overexpressing *Mt*L12 was a control that did not increase the MIC of IMB-T130. At the same time, streptomycin has equal antibacterial activity against the wild-type and overexpression strains. These results indicated that DHQS is probably the target of IMB-T130 and the series of compounds *in vivo*.

**Table 1:** Antibacterial activity of IMB-T130 against the *M. smegmatis* strains with a vector control or overexpressing plasmid for *Mt*DHQS and *Mt*L12 genes.

	Control vector (PMV261 plasmid)	Overexpressing *Mt*DHQS	Overexpressing *Mt*L12
IMB-T130	0.312 μg/ml	2.5 μg/ml	0.312 μg/ml
Streptomycin	0.156 μg/ml	0.156 μg/ml	0.156 μg/ml

### Intracellular anti-TB activity of IMB-T130 in macrophages

In a previous study, we reported that IMB-T130 has comparable anti-TB activity to rifampicin (RFP) and isoniazid (INH) for susceptible strains and it has a substantially lower MIC against drug-resistant strains because of its novel antibacterial mechanism^15^.

In this study, we tested the anti-TB activity of IMB-T130 in macrophages further in collaboration with Beijing Chest Hospital, Beijing, China. The intracellular anti-TB activity was tested using J774A.1 mouse monocyte macrophage and H37Rv (ATCC27294) strains^20^. The results showed that IMB-T130 could inhibit *M. tuberculosis* in macrophages in a dose-dependent manner. With 3 days exposure at 5 μg/mL, IMB-T130 treatment resulted in a 1.07-log_10_ reduction in *M. tuberculosis* colony forming units (CFU), and at a concentration of 10 μg/mL, a 1.36-log_10_ reduction was observed^21^. Treatment with RFP at 5 μg/mL led to a 1.84-log_10_ reduction in *M. tuberculosis* CFU (Table 2).

**Table 2:** Intracellular anti-TB activity of IMB-T130.

	Log_10_CFU					
	10 μg/mL	5 μg/mL	2 μg/mL	1 μg/mL	0.5 μg/mL	0 μg/mL
RFP		3.29 ± 0.12	3.74 ± 0.05		4.25 ± 0.07	
IMB-T130	3.77 ± 0.10	4.06 ± 0.03		4.85 ± 0.06		
Control						5.13 ± 0.03

## Discussion

The shikimate pathway involves seven enzymatic steps in the biosynthesis of chorismate product. Genomic studies have shown that this pathway is essential in bacteria, fungi, algae, parasites, and higher plants^22,23^. DHQS catalyses the second step of the shikimate pathway, which is conserved among various bacterial species^9,24^. Interestingly, a *Salmonella typhimurium* mutant with a DHQS knock-out displayed attenuated virulence in an animal model^25^. Importantly, the pathway is absent in mammals, which indicates that DHQS is a potential antibacterial drug target.

We measured the catalytic activity of DHQS quickly, simply, and accurately by measuring the free phosphoric acid released in the reaction using malachite green solution. IMB-T130, which was previously thought to be a multi-target compound, was identified as an excellent anti-TB agent^15^. In the present study, we found that IMB-T130 had a strong inhibitory effect on DHQS, suggesting that DHQS might be a more important target of IMB-T130.

The significant dose-dependent signal in the SPR experiments confirmed the interaction between IMB-T130 and DHQS, and the CD spectrum result indicated that IMB-T130 could bind to DHQS and induce changes in protein structure. In this study, HPLC-Q-TOF mass spectrometry was also performed to determine the molecular mass of the protein. The intact mass of DHQS did not increase after incubation with IMB-T130, which suggests the non-covalent binding of IMB-T130 to DHQS.

The interaction between IMB-T130 and DHQS was analysed by molecular docking using the DS 4.1 program. The docking model was initially based on the crystal structure of *Mt*DHQS without its substrate. The structure and function of DHQSs from different bacterial strains have been analysed, and it has been shown that DHQS is conserved in bacteria. Therefore, our model was optimized according to the crystal structure of *Sa*DHQS, which is similar to that of *Mt*DHQS. The molecular docking results suggested that five amino acids may be important in the interaction between DHQS and IMB-T130. These key amino acids were separately replaced with Ala to construct protein mutants. Leu30 and Val40 were also mutated to Ala to provide the controls. The resultant DHQS-M1–5 mutant proteins lost almost all of their catalytic activities, whereas DHQS-M6 and 7 retained most of their activities. The results showed that Lys144, Asn154, Asn246, His249, and Lys323 are essential for the function of *Mt*DHQS. We speculated that IMB-T130 binds to the active center of DHQS and inhibits its catalytic activity. The CD spectra of the mutant proteins showed that there were only slight differences compared with that of the original structure, indicating that the mutant proteins still maintained their secondary structure. However, small changes in the key sites could affect binding of the substrates, which reduced the catalytic activity.

Previous studies have shown that in bacteria, DHQS enzymes are structurally similar with highly conserved active sites. The catalytic capacity of DHQS is unusual in that it can perform multistep catalysis in one active site without the formation of unwanted by-products^19^. In this study, we showed that IMB-T130 can inhibit the catalytic activity of *Mt*DHQS, making it a potentially potent anti-TB agent. The MIC for IMB-T130 against the DHQS-overexpression strain was elevated, which confirms that the compound is likely to target DHQS. The results presented here improve our understanding of the catalytic mechanism of DHQS and provide opportunities to develop new agents targeting this enzyme, which could be an attractive target for the development of new drugs against *M. tuberculosis*. We also showed that IMB-T130 inhibits intracellular *M. tuberculosis* in a dose-dependent manner. Although its intracellular anti-TB activity is lower than RFP, considering its low toxicity against mammalian cells and high extracellular antibacterial activity, we believe that chemical modification of IMB-T130 will provide us with new lead compounds that will be highly effective against *M. tuberculosis*.

## Materials and Methods

### Materials

ATP, IPTG, and HEPES were purchased from Sigma-Aldrich (USA). The bacterial strains used for the MIC testing were purchased from the American Type Culture Collection (USA). Acid-albumin-dextrose-citric acid, and Middlebrook 7H9 and 7H10 medium were purchased from BD Biosciences (USA). DAHP (3-deoxy-D-arabinoheptulosonic acid 7-phosphate) was bought from Toronto Research Chemicals (Canada). Compound IMB-T130 was purchased from J&K Chemical (China; synthesized by Enamine Ltd., Ukraine). The malachite green solution (Biomol Green) was purchased from Enzo Life Sciences (USA). DMEM and fetal bovine serum were purchased from HyClone (USA). All the other chemicals used were of analytical grade.

## Methods

### Expression and purification of DHQS

*M. tuberculosis* H37Rv genomic DNA was provided by the Beijing Research Institute for Tuberculosis Control, China. The *aroB* gene was PCR-amplified using primers designed by Primer 5.0 software^26^. The sequences are 5′-GGCCGCCATATGACCGATATCGGCG-3′ (*Nde*I, sense) and 5′-TCAGCCCTCGAGTGGGGCGCAAACT-3′ (*Xho*I, anti-sense).

The PCR product was cloned into a pET30a vector using NdeI and XhoI restriction enzyme sites. The resulting pET30a-DHQS plasmid contained the DHQS gene fused to the 6 × His-tag at its C-terminal. The plasmid was transformed into *Escherichia coli* BL21 (DE3), and a single colony was picked and grown at 37 °C in LB medium containing 100 μg/mL kanamycin for cloning and maintenance. DHQS protein expression was induced in the cells by adding 0.2 μM IPTG with subsequent incubation at 25 °C for 12 h. The cells were collected and lysed by a high-pressure cracker. The target protein present in the supernatant was loaded on to a Ni^2+^ His Trap chelating column (GE Healthcare, USA) with a buffer (25 mM Tris, 500 mM NaCl, and 30 mM imidazole, pH 7.8). After that, the protein was eluted using a stepwise gradient of imidazole in the elution buffer (25 mM Tris, 500 mM NaCl, 350 mM imidazole, pH 7.8). The purified protein was analysed after separation by 12% (wt/vol) SDS-PAGE followed by Coomassie blue staining and western blotting with an anti-His antibody. The protein concentration was measured by the Bradford method using bovine serum albumin as a standard, and the purified DHQS protein was stored at −80 °C^15^.

### Enzyme assays and DHQS inhibitor screening

DHQS catalyse the conversion of DAHP to DHQ, and release a molecule of free phosphoric acid. The reaction was performed in a 50-µL volume with DHQS (1 μg/mL), NAD^+^ (40 μg/mL), DAHP (10 μg/mL), and Tris-HCl (50 mM, pH 7.5). The solution was added to a 96-well plate and incubated for 40 min at 37 °C. Then, 100 µL of malachite green solution was added and the absorbance of the solution was measured at 620 nm^27^.

We screened compounds with anti *M. smegmatis* activity for DHQS inhibitors. DHQS protein was pre-incubated with 0.5 μL of the test compound (10 μg/mL, dissolved in DMSO) at room temperature for 30 min in 49.5 μL of buffer (without DAHP); for the control group, 0.5 μL of DMSO was added. The reaction was initiated by adding 0.5 μL of DAHP solution followed by incubation (40 min, 37 °C). The absorbance was measured by a microplate reader. IMB-T130 showed strong inhibitory activity against DHQS. Hence, we measured the inhibitory effect of IMB-T130 on DHQS using the same assay. DHQS (1 μg/mL) was pre-incubated with increasing IMB-T130 concentrations (0.16–40 μg/mL) for 30 min at room temperature, with 1% DMSO used as a control.

### Confirmation of the interaction between IMB-T130 and DHQS by SPR assay and CD

An SPR experiment was performed to examine the interaction between DHQS and IMB-T130. Because the protein was unstable under acidic conditions, CM5 sensor chips were not suitable. Therefore, NTA sensor chips (BIAcore, GE Healthcare), which can capture the His-tag of the recombinant protein were used because they do not require an acidic buffering system. The sensor chips were first coated with Ni^2+^ before capturing the purified DHQS. Next, the chip was exposed to different concentrations of IMB-T130, and the response units for five different concentrations (1.875–30 μM) of IMB-T130 were measured by SPR (BIAcore X100 instrument, GE Healthcare).

CD is widely used to examine protein structures in solution. In the far UV spectral region (180–240 nm), the CD spectrum reflects the absorption of peptide bonds, and provides some information about the secondary structural features of a protein^18^. We determined the CD spectrum of the native DHQS and the complex of DHQS and IMB-T130.

### Protein model optimization and molecular docking between IMB-T130 and DHQS

The PDB crystal structure of the DHQS from *M. tuberculosis* with no substrate molecules in the crystal (PDB ID code: 3QBE) was used initially. We tried to generate the binding pocket automatically with Discovery Studio 4.1 (Chinese Academy of Medical Sciences and Peking Union Medical College, Beijing, China), but the model was too crude to perform the docking calculation. We found the crystal structure of DHQS from *S. aureus* complexed with Zn^2+^, NAD^+^, and the substrate analogue, carbaphosphonate (PDB ID code 1XAG) in the PDB data bank^28^. DHQS is conserved in many bacteria and fungi, such as *E. coli, Staphylococcus aureus*, and *Aspergillus nidulans*^10^. Therefore, we superimposed two protein models using the Discovery Studio 4.1 program to compare the spatial structures of these two homologous proteins. We also generated a protein sequence alignment between *S. aureus* DHQS (*Sa*DHQS) and *M. tuberculosis* DHQS (*Mt*DHQS) using Protein BLAST (https://blast.ncbi.nlm.nih.gov/Blast.cgi). The alignment between these proteins identified an overlapping 287-residue region with 35% sequence identity.

According to the crystal data for *Sa*DHQS, Asp130, Lys136, Asn146, Glu178, Lys181, Lys221, Arg235, Asn239, His242, His246, and Lys314 are important in the interaction between DAHP and the protein, and form the binding pocket of DAHP in *Sa*DHQS. Based on the protein sequence alignment, we found that the parallel amino acid residues in *Mt*DHQS are Asp138, Lys144, Asn154, Glu186, Lys189, Lys228, Arg242, Asn246, His249, His253, and Lys323. Because of the identical key amino acid residues and similar spatial structures, we speculated that *Mt*DHQS has the same binding pocket as SaDHQS. Thus, we defined the DAHP binding pocket in *Mt*DHQS based on these 11 key amino acids. The model is much more accurate than the initial model generated by the program automatically. IMB-T130 was docked in the active pocket using the CDOCKER program. The best results were obtained according to the scores of -CDOCKER energy and -CDOCKER interaction energy.

### Construction of mutant proteins and catalytic activity determination

Based on the molecular docking results and the alignment of several homologous protein sequences^10^, five amino acids, Lys144, Asn154, Asn246, His249, and Lys323, were identified as crucial in the interaction between the protein and IMB-T130, and in maintaining the function of DHQS. These amino acids are also conserved in *A. nidulans*, and participate in conformational changes within the DHQS active site^19^. These amino acids were separately replaced with Ala; Leu30 in the α1 helix, and Val40 in the β1 sheet were also mutated to provide controls. Mutations in pET30a-DHQS were created by site-directed mutagenesis using the Fast Mutagenesis System (TransGen Biotech, Beijing, China). Successful mutant plasmids were verified by DNA sequencing^29^. The catalytic activities of the mutants were determined using the same enzyme assay as was used for the wild-type enzyme.

### Pharmacokinetic studies in rats

Animal care and all experimental procedures were performed in accordance with local, national, and ethical principles and authority regulations, and were approved by the Institutional Animal Care and Use Committee of the Institute of Medicinal Biotechnology Institute. Sprague Dawley rats (226.5–261.2 g) were used in the pharmacokinetic studies. The formulation of IMB-T130 (4 mg/mL) was prepared in 5 mL water with 0.5% sodium carboxyl methyl cellulose for oral administration. For iv administration, 7.5 mg IMB-T130 was dissolved in 0.075 mL N-methyl pyrrolidone, and 0.6 mL of PEG400 and 0.825 mL of 20% hydroxypropyl-β-cyclodextrin (aq) were added. After vortexing for 5 min, the 5 mg/mL IMB-T130 formulation was prepared. Rats were dosed at 1 mL/kg by iv injection and at 5 mL/kg by oral gavage at 0 h. A 0.2 mL blood sample was drawn from the jugular vein at 5, 15, and 30 min, and 1, 2, 4, 6, 8, and 24 h. The blood samples were centrifuged (8000 rpm, 6 min) at 4 °C for plasma separation. The IMB-T130 plasma concentration was determined by liquid chromatography-mass spectrometry using an ACQUITY UPLC BEH C18 column (Waters; 1.7 µm, 2.1 × 50 mm)^30^.

### Determination of the MIC for *M. smegmatis* overexpressing DHQS

To overexpress the *Mt*DHQS protein in *M. smegmatis*, we used the plasmid pMV261-eGFP. The DHQS gene was inserted between the promoter and eGFP gene using BamHI and HindIII restriction enzyme sites so that DHQS was in frame with eGFP. The resulting pMV261:DHQS-eGFP plasmid was transformed into competent *M. smegmatis* cells. Recombinant protein expression was confirmed by fluorescence microscopy and western blotting with an anti-eGFP antibody. The MICs of IMB-T130 against *M. smegmatis* with a control vector (pMV261-eGFP plasmid), strains overexpressing a non-related gene (*MtL12*), and strains overexpressing DHQS-eGFP were determined by the conventional plate dilution method^31^.

### Evaluation of the intracellular anti-TB activity of IMB-T130

J774A.1 mouse monocyte macrophages were differentiated into adherent macrophages in 24-well tissue culture plates in DMEM with 10% fetal bovine serum. The plate was seeded with 4 × 10^5^ cells per well and incubated for 16 h at 37 °C with 5% CO_2_. *M. tuberculosis* was cultured for 3 weeks in 7H9 media containing 10% OADC and 0.05% Tween 80 at 37 °C with 5% CO_2_. The *M. tuberculosis* suspension was added to wells containing J774A.1 macrophages at a multiplicity of infection (MOI) of 5:1 after filtering with 8 μm filters. The plates were incubated for 4 h at 37 °C and 5% CO_2_, and then the media was removed and the cells were washed twice with PBS. Next, 2 mL fresh media (containing 10% fetal bovine serum) was added with different concentrations of drugs. The concentrations of IMB-T130 were 10, 5, and 1 μg/mL, while the concentrations of RFP were 5, 2.5, and 0.5 μg/mL. Control wells contained drug-free medium only. After culturing for 3 days, the media was removed. For *M. tuberculosis* CFU enumeration, infected macrophages were lysed with 200 μL 0.1% SDS at 37 °C. A series of dilutions in saline was made for spreading on 7H11C medium. Plates were incubated at 37 °C, 5% CO_2_ for 3–4 weeks before colony counting^21^.

## Electronic supplementary material

Supplementary materials
