# Supplementary material for: IMB-T130 targets 3-dehydroquinate synthase and inhibits Mycobacterium tuberculosis
Source: Sci Rep. 2018 Nov 28;8:17439. doi: 10.1038/s41598-018-35701-z (PMC6262012; doi:10.1038/s41598-018-35701-z)
Supplement: Supplementary file 1 — Supplementary materials [file 41598_2018_35701_MOESM1_ESM.pdf]

IMB-T130 targets 3-dehydroquinate synthase and inhibits *Mycobacterium tuberculosis*

Ningyu Zhu[1†], Xia Wang[1†], Dongsheng Li[1], Yuan Lin[2], Xuefu You[1], Jiandong Jiang[1, 2], Yanni Xu[1], Wei Jiang[1*‡], Shuyi Si[1*‡]

[1]Beijing Key Laboratory of Antimicrobial Agents, Institute of Medicinal Biotechnology, Peking Union Medical College and Chinese Academy of Medical Sciences, Beijing, 100050 China; [2]State Key Laboratory of Bioactive Substances and Function of Natural Medicine, Institute of Materia Medica, Chinese Academy of Medical Sciences and Peking Union Medical College, Beijing 100050, China

*Corresponding author: Tel: +86-10-63180604; Fax: +86-10-63180604; E-mail: sisyimb@hotmail.com

Supplementary materials

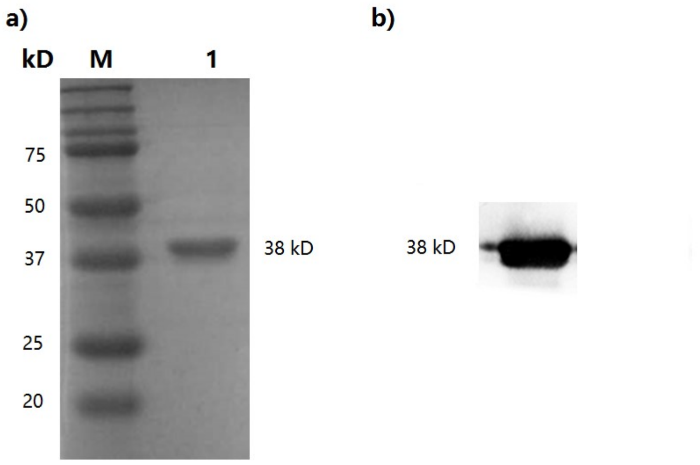

Supplementary Figure 1 Expression and purification of *M. tuberculosis* DHQS. a) Protein samples were separated by SDS-PAGE and the bands were visualised by Coomassie blue staining. Lane M, protein marker. Lane 1, purified His-tagged *Mt*DHQS. b) Expression of the His-tagged *Mt*DHQS protein detected by western blotting with an anti-His antibody.

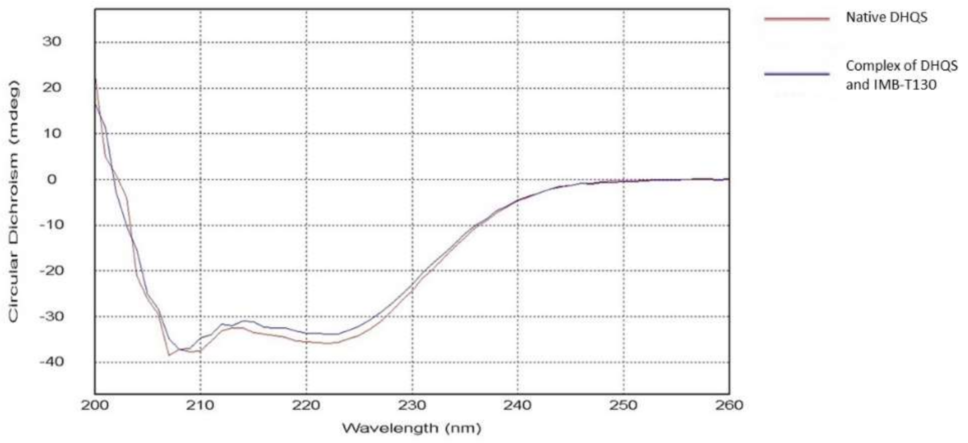

Supplementary Figure 2 Superimposed CD spectra of the native protein and DHQS complexed with IMB-T130.
